# COX-2 Silencing in Canine Malignant Melanoma Inhibits Malignant Behaviour

**DOI:** 10.3389/fvets.2021.633170

**Published:** 2021-08-26

**Authors:** Tatiany L. Silveira, Lisa Y. Pang, Alexandra Di Domenico, Emerson S. Veloso, Istéfani L. D. Silva, Helen L. Del Puerto, Enio Ferreria, David J. Argyle

**Affiliations:** ^1^Department of Pathology, Federal University of Minas Gerais, Belo Horizonte, Brazil; ^2^Royal (Dick) School of Veterinary Studies, Roslin Institute, The University of Edinburgh, Edinburgh, United Kingdom

**Keywords:** melanoma, COX-2, canine, malignant, one health

## Abstract

Metastatic melanoma is a very aggressive form of cancer in both humans and dogs. Dogs primarily develop oral melanoma of mucosal origin. Although oral melanoma in humans is rare, both diseases are highly aggressive with frequent metastases. This disease represents a “One Health” opportunity to improve molecular and mechanistic understanding of melanoma progression. Accumulating evidence suggests that cyclooxygenase-2 (COX-2) may play a critical role in the malignant behaviour of melanoma. In this study we analysed 85 histologically confirmed melanomas from canine patients and showed that COX-2 is overexpressed in both oral and cutaneous melanomas and that COX-2 expression correlates with established markers of poor prognosis. To determine the role of COX-2 in melanoma we developed two melanoma cell lines with stable integration of an inducible doxycycline-regulated expression vector containing a COX-2 targeted micro-RNA (miRNA). Using this system, we showed that cellular proliferation, migration and invasion are COX-2 dependent, establishing a direct relationship between COX-2 expression and malignant behaviour in canine melanoma. We have also developed a powerful molecular tool to aid further dissection of the mechanisms by which COX-2 regulates melanoma progression.

## Introduction

In humans, melanoma is one of the most aggressive types of cancer, every year killing ~50,000 people worldwide (1). Cutaneous forms represent the most common cases and are responsible for 65% of all skin malignancy-related deaths (2). Melanomas of the oral cavity are rarer but are associated with aggressive behaviour, including a tendency to repeated relapse and to metastasise, and poor patient outcomes (3). In contrast, the majority of melanomas forming in dogs are of mucosal origin, and most commonly seen in Scottish terriers, golden retrievers, poodles and dachshunds indicating a genetic predisposition to this disease. Canine oral melanoma is very aggressive and highly metastatic, with frequent metastases to local lymph nodes and lungs (4). Therefore, there is potential to adopt a “One Health” approach to improve molecular and mechanistic understanding of melanoma progression. Accumulating evidence suggests that cyclooxygenase-2 (COX-2) expression may be considered as a prognostic biomarker and as a potential therapeutic target in both human melanoma (5, 6) and canine melanocytic neoplasms (7).

COX-2, also known as prostaglandin-endoperoxide synthase 2 (PTGS2), is an inducible form of the rate-limiting enzyme in the metabolic conversion of arachidonic acid to prostaglandins, including prostaglandin E2 (PGE_2_), a significant mediator of inflammation and angiogenesis. Previously, COX-2 overexpression has been described in multiple human cancers including skin, bone, oesophageal, breast, lung, pancreatic, colon, cervical, prostate and bladder cancer, and is inversely associated with patient survival (8). At the molecular level the COX-2/PGE_2_ axis modulates a number of signal transduction pathways in human cancer cell lines that affect tumour cell proliferation, apoptosis, immune evasion, angiogenesis, cellular adhesion, differentiation and invasion (9). In canine osteosarcoma, we have previously shown that COX-2 expression is 141-fold higher in the cancer stem cell population compared to the non-cancer stem cell population, and that COX-2 plays a major role in tumour initiation (10). Consistently, inhibition of COX-2 with either non-steroidal anti-inflammatory drugs (NSAIDs) or with specific COX-2 inhibitors (COXIBs) can reduce the mortality of several types of cancer (11) and can switch the immune response from a tumour-promoting profile to a tumour-destructive one, re-ordering the tumour microenvironment (12). However, despite ample epidemiological evidence supporting an inverse relationship between NSAID use and the incidence and progression of cancer the clinical application of these drugs for cancer prevention remains controversial. NSAIDs and COXIBs have been shown to have off-target effects and to supress the biosynthesis of other physiologically relevant prostaglandins that are associated with the adverse side-effects of these drugs on the GI tract and cardiovascular system, including elevated risk of myocardial infarction and stroke (13, 14). We have previously shown that mavacoxib (Trocoxil™), a clinically relevant COXIB in veterinary medicine, which is currently licenced to treat pain and inflammation in canine osteosarcoma, can exert anti-tumourigenic effects on a panel of canine cancer cell lines with low COX-2 expression indicating that this drug may act in a COX-2 independent manner (15).

In the context of canine melanoma, the exact role of COX-2 in the progression of the disease has not been fully elucidated. Previous studies have examined the association between COX-2 expression and prognostic factors, such as Ki-67 proliferation index and survival time after diagnosis, in melanocytic tumours and concluded that COX-2 may be associated with malignancy and may be a potential prognostic marker (7, 16, 17). *In vitro*, COX-2 inhibition by celecoxib in two canine melanoma cell lines has been shown to supress cell growth and have anti-tumour effects (18). One of the limitations of veterinary research is a lack of molecular tools to dissect the contribution of COX-2 to tumour progression. A model system in which reversible loss of COX-2 function could be controlled in space and time would be of significant value. In this study we have correlated the expression level of COX-2 in samples from canine patients with established markers linked to poor prognosis, and we describe the development of two melanoma cell lines with stable integration of a doxycycline-regulated expression vector containing a COX-2 targeted micro-RNA (miRNA), to enable us to reversibly suppress endogenous *COX-2* gene expression. We utilised this system to show that COX-2 is an essential driver of cellular proliferation, migration and invasion of canine melanoma cells.

## Materials and Methods

### Retrospective Case Material and Histologic Classification

Eighty-five cases from canine patients in which melanoma was diagnosed were retrieved from the archive of the Comparative Pathology Laboratory, Department of Pathology, Universidade Federal de Minas Gerais, Belo Horizonte, Brazil. Informed client consent was obtained for all patient samples used in this study. Of these, 29 cases were oral melanomas and 56 were cutaneous melanomas. To confirm the primary diagnosis each sample was re-examined independently by two pathologists (E.F. and T.S.) according to the criteria, established by the World Health Organisation (WHO), of the International Histological Classification of Tumours of Domestic Animals (19), using a Olympus BX41 microscope. Formalin-fixed and paraffin-embedded (FFPE) tissues were cut into sections (4 μm) using a Leica RM2245 microtome. Deparaffinised samples were used for Hematoxylin and Eosin (H&E) staining. Clinicopathological features were evaluated: (1) presence of ulceration; (2) degree of pigmentation; (3) morphology of neoplastic cells (spindle, epithelioid or mixed); (4) mitotic index (MI); and (5) tumoural vascular invasion. The MI was determined by counting all the mitoses within 10 random, non-overlapping high-power fields (HPFs) ( ×400), FN 22/40x objective (2.37 mm^2^) (20). A MI was attributed according to the number of mitoses: low MI (<4 mitoses per 10 HPFs); high MI (≥4 mitoses per HPFs) (21). The degree of pigmentation was estimated using a subjective scale from 0 (no pigmentation), 1 (pigmentation in 25% of neoplastic cells), 2 (pigmentation in 25–50% of neoplastic cells) to 3 (pigmentation in >50% of neoplastic cells).

### Immunohistochemical Analysis

FFPE tissue samples were cut into 4 μm sections and processed for immunohistochemical (IHC) analysis of S-100, melan-A, PNL-2, COX-2 and Ki-67. All immunostainings utilised the streptavidin-biotin-peroxidase complex method with a commercial detection anti-mouse/anti-rabbit system (Novolink Polymer Detection Sistem; Leica Biosystems, Newcastle upon Tyne, UK) according the manufacturer's instructions. Antigen retrieval was performed by Pascal® in citrate buffer pH 6.0 (DakoCytomation Target Retrieval Solution) at 125°C for 2 min, followed immediately by 20 min at room temperature (RT). All sections were incubated with the primary specific antibody: S-100 (1:100 dilution, Clone S100, Dako, Glostrup, Denmark), melan-A (1:100 dilution, Clone A103, Dako, Glostrup, Denmark), Melanoma Antigen (1:100 dilution, Clone PNL-2, Santa Cruz Biotechnology, Dallas, Texas, USA), COX-2 (1:80 dilution, Clone SP21, Thermo Fisher Scientific, Walthan, MA, USA) or Ki-67 (1:50 dilution, Clone MIB-1, Dako, Glostrup, Denmark) for 18 h at 4°C. The antibody reactions were visualised with chromogen 3,3′ -diaminobenzidine tetrachloride (DAB) diluent (Dako, Code K3468), incubated for 3 min at RT. The samples were washed 3x times in distilled water for 5 min at RT, then counterstained with Giemsa for 30 min at RT, dehydrated and mounted (22). Sections treated with isotype-matched primary antibodies mouse anti-human IgG were used as negative controls and this study also included adequate positive controls: canine mammary tumour for COX-2; and canine epidermis for melan-A, PNL-2, and Ki-67. Images were captured on BX41 Olympus microscope. A subjective scale was then used to establish the degree of positive staining for S-100, melan-A and PNL-2 (23, 24). For COX-2 staining a distribution score and intensity were multiplied to obtain a total score, which ranged from 0 to 12. Distribution was estimated as the percentage of positive cells in 10 HPFs (x400): 0 (absent); 1 (<10% stained cells); 2 (10–30% stained cells); 3 (>30–60% stained cells); and 4 (>60% stained cells). For staining intensity: 0 (absent); 1 (weak); 2 (moderate); and 3 (strong). The samples with a final score 0–5 were considered to have COX-2 low expression, and samples with a score of 6–12 were considered to have high COX-2 expression (24). The Ki-67 index was evaluated by counting 500 tumour cells within a microscopic grid at high magnification ( ×400) and the index was expressed as a percentage.

### RNA Extraction and Real-Time Quantitative PCR From FFPE Samples

RNA was extracted from FFPE tissue samples using the RecoverAll™ Total Nucleic Acid Isolation Kit (Thermo Fisher Scientific, Walthan, MA, USA, Code AM1975), according to the manufacturer's instructions. Extracted RNAs were quantified and 260/280 nm absorbance determined by NanoDrop Nucleic Acid Quantification (Thermo Fisher Scientific, Walthan, MA, USA). Total RNA was reverse transcribed using the M-MLV kit (Invitrogen, CA, USA, Code 280250-13) according to the manufacturer's instructions. The RNA integrity was evaluated by conventional RT-PCR analysis with the amplification of a 120 base pair fragment of the dog GAPDH reference gene, using the forward 5′-TTCCACGGCACAGTCAAG-3 and reverse 5′-ACTCAGCACCAGCATCAC-3′ primers. For quantitative real-time PCR 90 ng of cDNA was used in a final qPCR reaction with SYBR Green® PCR Master Mix Kit according to manufacturer's instructions (Invitrogen, CA, USA) and ABI PRISM® 7500 Sequence Detection System (Carlsbad, CA, USA). For the RT-qPCR the cycling conditions used were: (stage 1) 1 cycle of 50°C for 2 min; (stage 2) 1 cycle at 95°C for 10 min.; (stage 3) 40 cycles of 95°C for 15 s then 55°C for 1 min. After completion of the amplification reaction, melt-curve analysis was carried out at 95°C for 15 s to produce a dissociation curve. Relative gene expression levels were obtained by normalisation to the expression level of a housekeeping gene (HPRT) (25). Primer sequences are shown in Table 1.

**Table 1:** Primer sequences for the amplification of qRT-PCR products from canine FFPE tissue samples.

**Gene**	**Forward primer (5^**′**^-3^**′**^)**	**Reverse Primer (5^**′**^-3^**′**^)**
COX-2	CCTGACACCTTGCAAATAG	ATTCCACAAACTGGGTAAGG
HPRT	CCTTGGTCAAGGAGCATAATC	GTCAAGGGCATATCCTACAAC

### Cell Culture

The panel of canine melanoma cell lines used in this study included CMGD2, CMGD-5, CML-10 and TLM1 (Kerafast, Boston, MA, USA), cultured in Dulbecco's modified Eagle's medium (DMEM) supplemented with 10% foetal bovine serum, 100 μg/ml streptomycin (ThermoFisher Scientific) and 5% HEPES (ThermoFisher Scientific). Canine transitional cell carcinoma of the urinary bladder cell line (K9TCC) (a kind gift from Deborah Knapp and Jane Stewart, Purdue University) was grown in DMEM/F-12 (ThermoFisher Scientific) containing HEPES and L-glutamine and supplemented with 10% FBS and 100 μg/mL penicillin/streptomycin. All cells were maintained at 37°C in a humidified atmosphere with 5% CO_2_.

### Real-Time Quantitative PCR

RNA was extracted from cells using the RNeasy Kit (Qiagen, CA USA) according to the manufacturer's instructions. Real-time PCR was executed using 50 ng of amplified RNA and the Stratagene Mx3000p qPCR system (Aligent, CA, USA), using the Platinum® SYBR® Green qPCR SuperMix-UDG according to manufacturer's instructions (Invitrogen, CA, USA). For the RT-qPCR the cycling conditions used were: (stage 1) 1 cycle of 95°C for 2 min; (stage 2) 40 cycles of 95°C for 5 s followed by 60°C for 10 s. After completion of the amplification reaction, melt-curve analysis was carried out according to the instrument's instructions. Relative gene expression levels of COX-2 were obtained by normalisation to the expression level of a housekeeping gene (GAPDH) (25). Primer sequences are shown in Table 2.

**Table 2:** Primer sequences for the amplification of qRT-PCR products from canine cell lines.

**Gene**	**Forward primer (5^**′**^-3^**′**^)**	**Reverse Primer (5^**′**^-3^**′**^)**
COX-2	GCCCTATACATCATTCGAAGAAC	CACCATAAAGGGCCTCCAA
GAPDH	GGGAAGATGTGGCGTGAC	GAAGGCCATGCCAGTGAG

### Construction of a Canine Inducible COX-2 miRNA Expression Vector

The generation of inducible vectors expressing COX-2 miRNAs were constructed using custom designed complementary canine COX2 oligos (Invitrogen's RNAi Designer, USA), with each containing 4 nucleotide overhangs necessary for directional cloning. The complementary COX2 oligos were annealed and the resulting double-stranded oligo was cloned into pcDNA™6.2-GW/EmGFP-miR vector (pcDNAGFPmiRNACOX2-346) according to the Block-iT ™ PolII miR RNAi expression vector kit manual (Invitrogen). A negative control plasmid, pcDNATM6.2-GW/EmGFP-miRneg (included in the kit), containing a miRNA insert predicted not to target any known vertebrate gene, was also used for the subsequent cloning steps.

Custom designed canine double-strand COX2-346 oligo, with 4 nucleotide overhangs (bold), cloned into pcDNA™6.2-GW/EmGFP-miR vector:

**TGCTG**TGGACTCTCAATCAAATGTGAGTTTTGGCCACTGACTGACTCACATTTTTGAGAGTCCA
CACCTGAGAGTTAGTTTACACTCAAAACCGGTGACTGACTGAGTGTAAAAACTCTCAGGT**GTCC**

The pcDNAGFPmiRNACOX2-346 and pcDNA™6.2-GW/EmGFP-miRNeg vectors are Gateway® compatible due to the presence of attB sites flanking the COX-2 miRNA insert and can be transferred to other Gateway® adapted destination vectors that contain attR sites. After removal of emGFP, canine COX-2 miRNAs and miRNeg expression cassettes were transferred to the pTRIPZdest destination vector (a kind gift from Dr Peter Hohenstein, Roslin Institute) using the rapid Invitrogen BP/LR protocol (Invitrogen). The destination vector pTRIPZdest is a lentiviral inducible vector engineered to be Tet-On. Following transfer of the COX2miRNA in the pTRIPZdest, the tetracycline response element (TRE), placed upstream of a minimal promoter, will drive the co-expression of a TurboRFP reporter gene and the COX2miRNA if the transactivator (rtTA3) binds to TRE which only occurs in the presence of doxycycline (DOX). The induced expression of TurboRFP allows tracking of transfection efficiency, TRE promoter activity and COX2miRNA expression. The pTRIPZdestmiRCOX2 vectors contain the puromycin resistance gene (puro) which allows for puromycin selection of any mammalian cell lines that are stably transfected with the vectors.

### Transfection of Inducible COX-2 miRNA Expression Vector and the Generation of Stable Cell Lines

CMGD-2, CMGD-5, CML-10 and TLM-1 cells were seeded at 2 ×10^5^ in 6-well plates and incubated for 24 h. At 80% confluency cells were transfected with 2.5 μg DNA (pTRIPZdestmiRCOX2-346 expression vector) per well at a final concentration of 1 μg/μl using 12.5 μl (CMGD-2 and CMGD-5), 9 μl (CLM-10) and 6 μl (TLM-1) Lipofectamine® 2000 reagent (ThermoFisher Scientific, USA) according to the manufacturer's instructions. pTRIPZdestmiRNeg plasmid negative control plasmid was used as a negative control in subsequent experiments. To establish stable transfection, cells were exposed to 2 μg/ml of puromycin 48 h post-transfection. After 10–14 days of selection, puromycin-resistant colonies were pooled and expanded.

### Proliferation Assay

Transfected cells were seeded in triplicate in opaque 96-well plates (Corning, CA, USA) at 2,000 cells/well. Cells were incubated 37°C, 5% CO_2_ and cell viability was assayed 48 h later using the CellTiterGlo® Luminescent Cell Viability Assay (Promega, Madison, USA, Code G7570) according to the manufacturer's instructions. Luminescence was recorded using a Viktor3 luminometer (PerkinElmer, Massachusetts, USA). Data was averaged, and then normalised against the average signal of untreated/negative control treated samples.

### Colony Formation Assay

Transfected cells were seeded at 500 cells/10 cm plate and immediately treated with the indicated dose of DOX. Plates were incubated until colonies were visible at 37°C in a humidified CO_2_ incubator. Growth media was changed approximately once a week. The colonies were fixed by incubating for 5 min with ice-cold methanol at RT. Colonies were air dried and then stained with Giemsa (Invitrogen, Paisley, UK) for 20 min at RT. The total number of colonies were counted.

### Invasion Assay

Invasion assays were performed using transwell system with two compartments separated by 8 μm pore polycarbonate philtres coated with Matrigel (BD Biosciences). In the upper compartment ~2 ×10^4^ cells were seeded in serum-free media, and the lower compartment contained media spiked with 5% FBS. Cells were allowed to transmigrate for 24 h. Non-migrated cells on the upper side of the transwell were removed. Cells on the underside of the transwell were fixed with methanol and stained with 0.1% crystal violet. These represented cells that had migrated through the philtre. Ten different fields (x200) were counted in triplicate experiments.

### Cellular Migration Assay

Transfected cells were incubated at 37°C in humidified 5% CO_2_ incubator grown until 80–90% confluent in 6-well plates. A scratch was made in the confluent monolayer of cells with a pipette tip. The gap was visualised using the Axiovert 40 CFL microscope coupled with an AxioCAM HRm camera (Carl Zeiss Ltd, Cambridge, UK) and measured at 6 points/gap and an average taken. Measurements were made over the indicated time course. Scratch width was converted to “relative migration distance” of cells, where the distance is a percentage of the initial wound width.

### Statistical Analysis

Data were expressed as a mean ± S.D. Statistical analysis was performed with GraphPad Prism version 5.0 for Windows (GraphPad software, CA USA) using Spearman's correlation for non-parametric analysis test, analysis of variance and Student's *t*-test or mann-whitney test. The criterion for significance was *p* < 0.05 for all comparisons.

## Results

### Immunohistochemical Analysis of Canine Melanoma Samples

A total of 85 melanoma tumours (29 orals and 56 cutaneous) were included in this study and classified accordingly: 23 spindle cell, 53 epithelioid cell and nine mixed. High pigmentation level is inversely correlated with overall survival time in human patients and is an important marker of the disease (26). Here the degree of pigmentation was estimated using a subjective scale from 0 (no pigmentation) to 3 (pigmentation in >50% of neoplastic cells). Of the 85 samples: 20 had no pigmentation; 42 scored 1; 7 scored 2; and 16 scored 3. Regarding the mitotic index (MI), 74 cases were evaluated (24 orals and 50 cutaneous), of those, 14 oral and 20 cutaneous melanomas had a high MI (≥4 mitoses per 10 HPF) (Table 3). The MI could not be evaluated on 11 other cases due to high pigmentation.

**Table 3:** Clinicopathological parameters of the oral and cutaneous melanomas.

	**Oral**	**Cutaneous**
**Histological type**		
Epithelioid cells	23	30
Spindle cells	3	20
Mixed	3	6
**Pigmentation**		
0 (no pigmentation)	12	8
1 (25% of cells)	12	30
2 (25–50%)	1	6
3 (>50%)	4	12
**Mitotic index**		
<4 mitoses	10	30
≥4 mitosis	14	20

**Figure 1:**
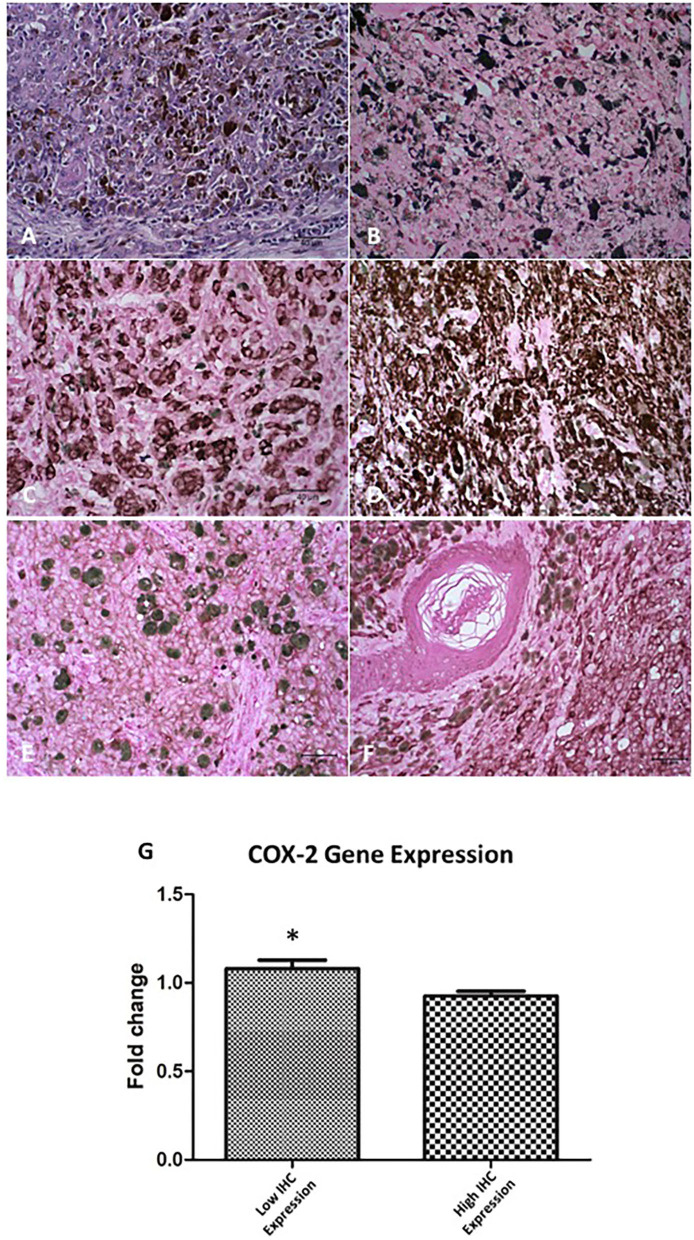
COX-2 expression in oral and skin melanoma in the dog. **(A)** Oral, mucosal melanoma, no pigmentation. H&E staining. **(B)** Skin. Ki-67 staining in the nucleus of neoplastic cells. DAB staining of chromogen. Giemsa counterstain. **(C)** Oral. Melan-A staining in the cytoplasm of neoplastic cells. DAB staining of chromogen. Giemsa counterstain. **(D)** Skin. Melanoma Antigen (PNL-2) staining in the cytoplasm of neoplastic cells. DAB staining of chromogen. Giemsa counterstain. **(E)** Oral. COX-2 staining, low expression (score 4) in neoplastic cells. DAB staining of chromogen. Giemsa counterstain. **(F)** Skin. COX-2 staining, high expression (score 12) in neoplastic cells. DAB staining to chromogen. Giemsa counterstain. All images are objective 40x. Scale bar represents 40 μM. **(G)** Relative COX-2 mRNA expression comparing IHQ samples with low and high COX-2 immunostaining (*p* = 0.02*).

The immunoreactivity for S-100, melan-A, and PNL-2 was observed as brown cytoplasmic staining. All samples were positive for S-100 protein and PNL-2, and 75 of 85 were positive for melan-A. Of the 10 cases that were negative for melan-A the diagnosis was confirmed by positive staining for PNL-2 (Table 4). Overall, a high COX-2 expression revealed a statistically significant correlation with Ki-67 (*p* = 0.03), however when oral and skin melanomas were analysed independently only the skin tumours presented statistical significance (*p* = 0.01) (representative images of Ki-67, melan-A, PNL-2 and COX-2 staining are shown in Figure 1).

**Table 4:** Immunohistochemical positivity of oral and cutaneous melanomas by S-100, melan-A, PNL-2, and COX-2 antibodies.

**Molecular markers**	**Oral**	**Cutaneous**
S-100	100% (29/29)	100% (56/56)
melan-A	93% (27/29)	100% (56/56)
PNL-2	100% (12/12)	100% (8/8)
**COX-2**		
Score 0	7% (2/29)	12% (7/56)
Score 1–5	59% (17/29)	45% (25/56)
Score 6–12	34% (10/29)	43% (24/56)

We found that COX-2 was located in the cellular membrane, cytoplasm, and nuclear membrane in a diffuse and homogeneous pattern. In the primary tumour the labelling intensity of COX-2 was comparable to that of neoplastic intravascular emboli, however, the extension of COX-2 staining in neoplastic intravascular emboli was always diffuse. These results confirmed that COX-2 was expressed, at some degree, in 90% of all tumours examined (Figures 1E,F) and that 34 of 85 of these tumours had a combined distribution and intensity score of 6–12. Of the 29 oral melanomas, 10 showed a COX-2 score of 6–12, whereas 24 of 56 of cutaneous melanomas scored 6–12 (Table 4).

The high COX-2 expression revealed a statistically significant correlation with both oral and cutaneous tumours with Ki-67 (*r* = 0.45; *p* = 0.03), MI (*r* = 0.49; *p* < 0.001), lymphatic invasion (*r* = 0.41; *p* = 0.04) and the histological type of tumour (epithelioid and mixed) (*r* = 0.51; *p* = 0.003). In the cutaneous tumours the presence of skin ulceration (*r* = 0.49; *p* = 0.02) was also statistically correlated with high COX-2 levels. Thus, these results suggest that high COX-2 protein levels could be associated clinicopathological aggressiveness parameters. To correlate this data to COX-2 gene expression we isolated RNA from the FFPE samples and determined COX-2 gene expression by qRT-PCR. The samples were divided according to the previous IHC results into low and high COX-2 protein levels. The gene expression revealed a statistically significant inverse correlation with the COX-2 protein expression (*r* = 0.64; *p* = 0.0008) (Figure 1G).

### Construction of an Inducible COX-2 miRNA Expression Vector

**Figure 2:**
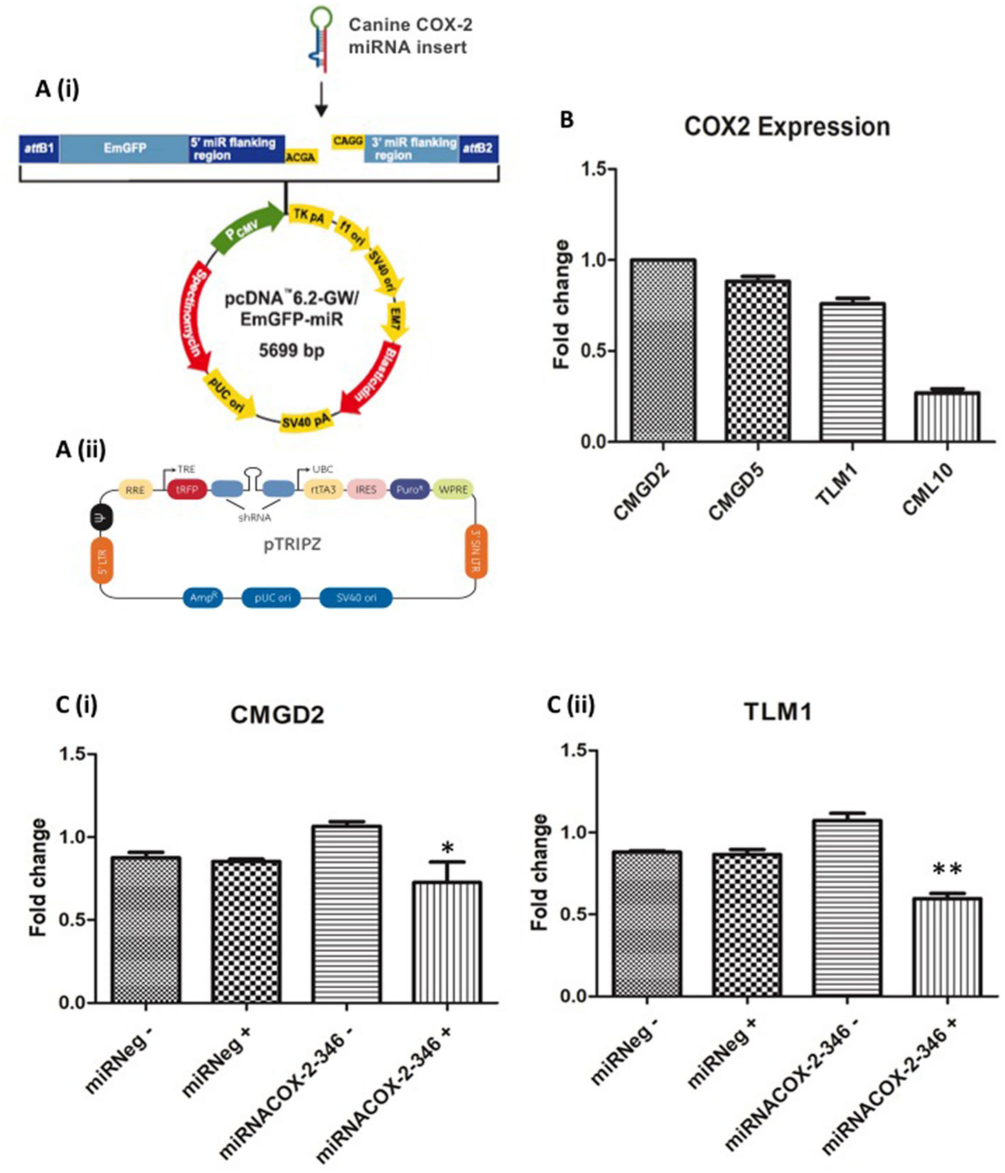
Construction of an inducible COX-2 miRNA expression vector in canine melanoma cell lines. **(A)** Canine double-stranded COX-2 miRNAs were generated and cloned into (i) pcDNA™6.2-GW/EmGFP-miR vector (Invitrogen) and transferred to (ii) pTRIPZdest destination vector. **(B)** Basal expression level of COX-2 in CMGD2, CMGD5, TLM1, and CML10 canine melanoma cell lines. **(C)** Validation of inducible COX-2 mi-RNA expression: (i) CMGD2 cells and (ii) TLM1 cells were transfected with 2.5 μg of the vector or negative control after 24 h cells were treated with 1 μg/ml DOX (+) or vehicle control (–) and COX-2 expression was determined by qRT-PCR. Data shown as fold change relative to miRneg (+) expression (*p* = 0.03*, *p* = 0.002**).

Artificial miRNAs were engineered to have 100% homology to the target COX-2 sequence and result in cleavage. To test the candidate miRNAs for suppression of COX-2 expression, three canine COX-2miRNA vectors were transiently transfected by lipofection into a canine bladder cancer cell line (K9TCC) that highly expresses COX-2. The vector contains a CMV promoter-driven puromycin resistance gene and a green fluorescent protein (GFP) gene to select and identify transfected cells. EmGFP/COX-2miRNA and EmGFP/miRNeg expressing cells were sorted 48 h post-transfection by fluorescence-activated cell sorting (FACS). COX-2 down-regulation was assessed by qRT-PCR using the comparative CT (2ΔΔCT) method. The pcDNAemGFPmiRCOX2 vectors are Gateway® compatible (Figure 2A(i)). After removal of emGFP, canine COX-2miRNAs and miRNeg expression cassettes were transferred to the pTRIPZdest destination vector (a kind gift from Dr Peter Hohenstein, Roslin Institute) (Figure 2A(ii)). This is an inducible system activated by DOX. COX-2 down-regulation was assessed by qRT-PCR in K9TCC inducible clones grown over a week in DOX (2 μg/ml) (Data not shown).

### Generation and Characterisation of Melanoma Cell Lines Stably Expressing an Inducible COX-2 miRNA Vector

The basal gene expression of COX-2 in each cell line was determined by qRT-PCR. COX-2 expression was highest in CMGD2, whereas the lowest level COX-2 expression was detected in CML10 cells (Figure 2B). The CMGD2 and TLM1 melanoma cell lines were stably transfected with the pTRIPZdestmiRCOX2-346 vector and control cells with the pTRIPZdestmiRNeg plasmid. Puromycin was applied 48 h post-transfection to select for stable expression of the plasmid. CMGD2 and TLM1 puromycin-resistant colonies were pooled 10–14 days following transfection, expanded and frozen for subsequent experiments. To test the inducible system, CMGD2 and TLM1 puromycin-resistant pools expressing the pTRIPZdestmiRCOX2-346 (miRNACOX2-346) vector or miRNeg control vector were exposed to 1 μg/ml of DOX. The pTRIPZdestmiRCOX2-346 vector contains a tetracycline-dependent promoter which in the presence of DOX drives the expression of the miRNA and the expression of a TurboREP reporter, allowing assessment of promoter activity. Here TurboRFP expression was assessed by fluorescence microscopy. We confirmed that COX-2 gene expression was decreased in CMGD2 cells expressing miRNACOX2-346 +DOX and TLM1 cells expressing miRNACOX2-346 +DOX compared to cells expressing the miR negative control (Figure 2C(i) and (ii), respectively).

### Effects of COX-2 Knockdown on Cell Proliferation

**Figure 3:**
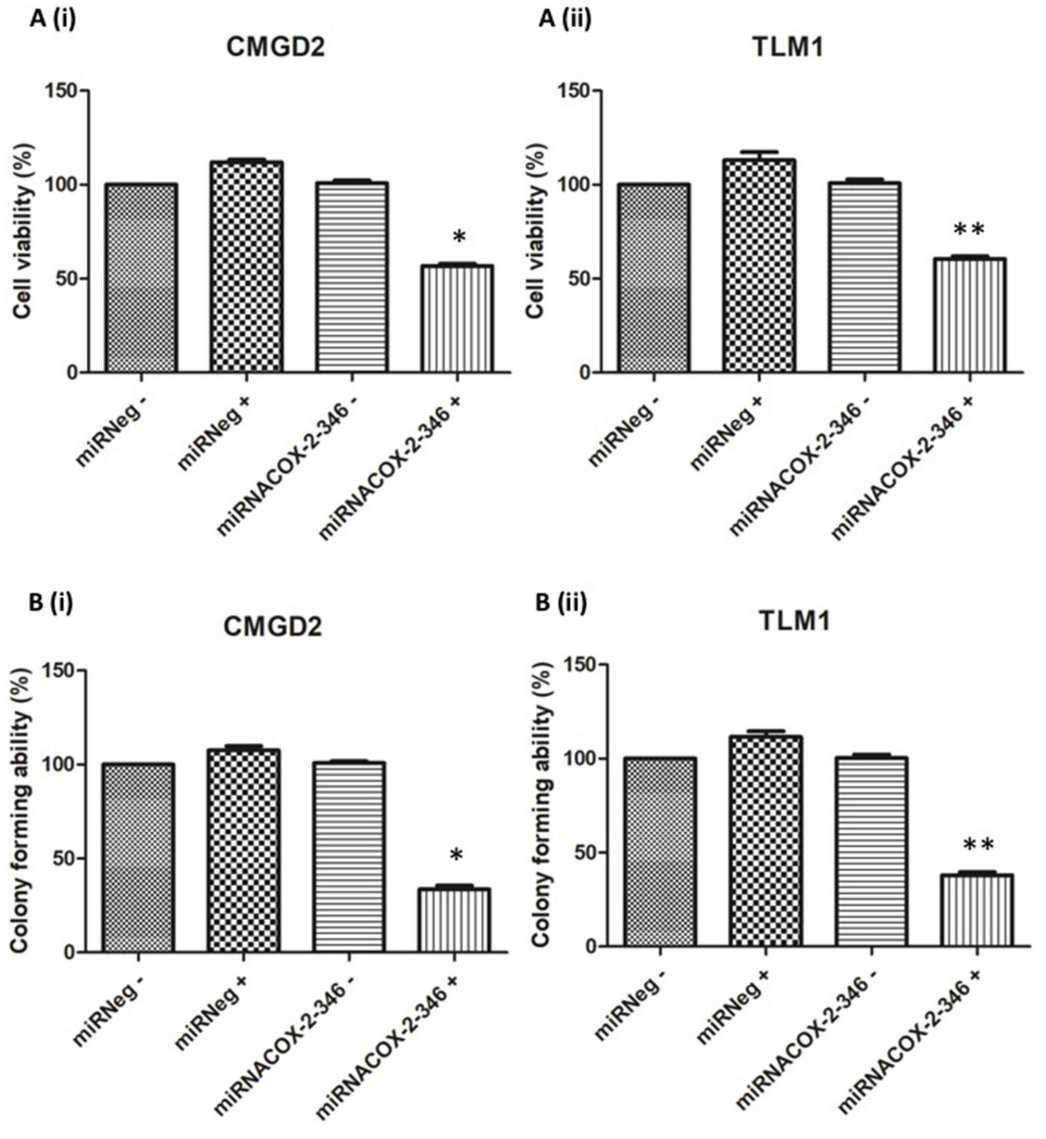
Melanoma cell viability and colony-forming ability is COX-2 dependent. **(A)** Cell viability of CMGD2 (i) and TLM1 (ii) was determined after transfection with either miRNACOX2-346 or the miRneg negative control with (+) or without (–) DOX. Cell viability was assayed 48 h after DOX treatment (*p* = 0.001*, *p* = 0.003**). **(B)** Colony forming ability of CMGD2 (i) and TLM1 (ii) was determined after transfection with either miRNACOX2-346 or the negative control with (+) or without (–) DOX (*p* = 0.0001*, *p* = 0.01**).

To determine if COX-2 has an effect on cell viability of melanoma cell lines we used the CMGD2 and TML1 cell lines stably expressing the miRNACOX2-346 or the miR negative control. Here we induced knock-down of COX-2 by treating cells with 1 μg/ml DOX (+) or water (–) as a vehicle control. Cell viability was assayed 72 h after treatment. Here we showed that cell viability is COX-2 dependent in both CMGD2 (Figure 3A(i)) and TML1 cell lines (Figure 3A(ii)). Clonogenic survival analysis, as a measure of cell reproductive death after treatment, shows that only cells expressing the miRNACOX2-346 vector and treated with DOX have a reduction in colony-forming ability (Figure 3B). Taken together, these results indicate that COX-2 may be an important driver of melanoma cell survival.

### Invasiveness and Migration Are COX-2 Dependent

**Figure 4:**
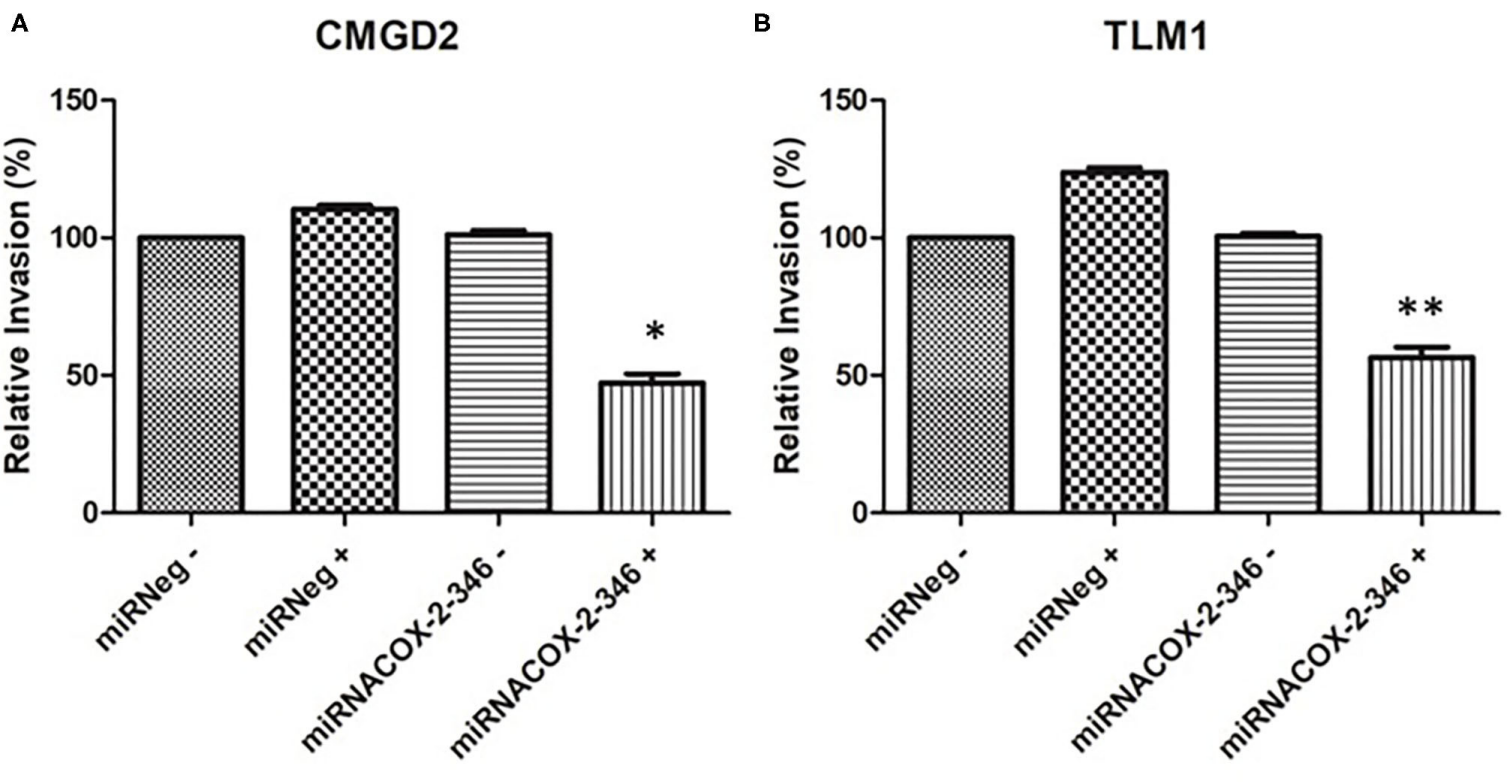
COX-2 regulates cell invasion. **(A)** CMGD2 and **(B)** TLM1 cells were transfected with either miRNACOX2-346 or the miRneg negative control with (+) or without (–) DOX. Invasion potential was determined 48 h after seeding into a transwell chamber assay (QCM™ collagen-based cell invasion assay kit) (*p* = 0.0004*, *p* = 0.0002**).

**Figure 5:**
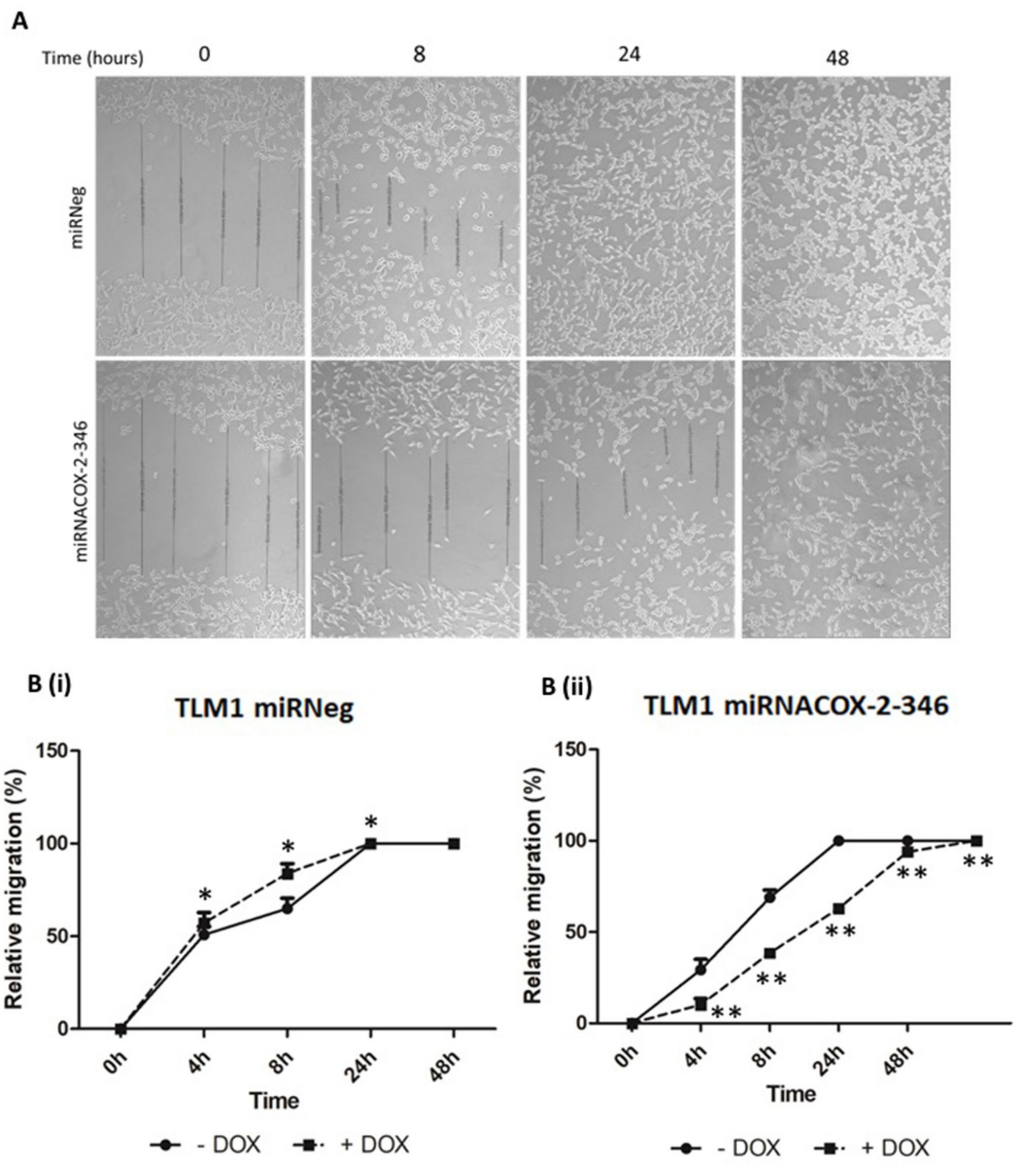
Migration of canine melanoma cells is COX-2 dependent. **(A)** Representative image of a scratch assay of TML1 cells transfected with either miRNACOX2-346 or the miRneg negative control, treated with 1 μg/ml DOX. After 24 h incubation a scratch was made and the gap distance was measured at regular intervals until the gap closed. **(B)** Quantification of this data (i) miRneg negative control and (ii) miRNACOX2-346 (*p* < 0.05*, *p* < 0.0001**).

The invasive capacity of CMGD2 and TLM1 cells stably expressing miRNACOX2-346 was determined using a Boyden chamber assay. CMGD2 miRNACOX2-346 and TLM1 miRNACOX2-346 cells treated with 1 μg/ml DOX (+) showed a significant reduction in invasiveness compared to both the vehicle control and the miRNeg control (Figures 4A,B). Similarly, we assessed migration potential using a standard scratch assay and showed that induced silencing of COX-2 reduced the ability of both TML1 (Figure 5) and CMGD2 (data not shown) to close the wound. These results indicate that COX-2 enhances the migration and invasion capabilities of canine melanoma cells.

## Discussion

The COX-2 pathway is the evolutionarily conserved master switch that activates the inflammatory response. Under normal conditions, acute inflammation is a short-term, self-limited process activated by an inflammatory stimulus. However, tumours covet a level of “smouldering” inflammation within their microenvironment and as such over-expression of COX-2 is a prominent feature of several human cancers, and has been associated with tissue re-modelling, angiogenesis, cancer cell survival, metastasis, and immune evasion (8). In human melanoma COX-2 expression has been linked to malignant behaviour. In this study we examined the relationship between COX-2 expression and canine melanoma. Here we present evidence that COX-2 is overexpressed in both oral and cutaneous melanomas and that this is related to the degree of pigmentation, MI, Ki-67 proliferation index and the expression of validated melanoma markers including S-100 and melan-A. We also developed a novel inducible COX-2 miRNA vector to effectively knock-down COX-2 expression in a panel of canine melanoma cell lines, and showed that a number of aggressive phenotypes are COX-2 dependent.

Our data is consistent with the notion that canine melanoma is an effective model for comparative oncology. Spontaneous melanocytic neoplasms arise in many domestic animals including dogs, cats, horses and pigs. However, malignant melanoma is more common in the domestic dog than other species and a majority of cases arise in either the oral cavity or the skin (4). Canine oral melanomas are highly aggressive and frequently metastasise, principally to local lymph nodes and lungs, whereas cutaneous melanomas are most often benign. In contrast to dogs, cutaneous melanomas are more common in humans, and melanoma of the oral cavity is extremely rare but exceedingly aggressive, metastatic and associated with poor patient outcomes (27). Because of their rareness, accounting for <1% of all melanomas in humans, there is currently a lack of knowledge regarding pathogenesis including etiological factors, risk factors and molecular drivers (28). We propose that there is a significant opportunity to utilise the dog model to improve molecular and mechanistic understanding of melanoma progression.

Previously, overexpression of COX-2 has been correlated with the development and progression of human cutaneous melanoma (29, 30) and has been proposed as a prognostic marker. In oral melanoma, COX-2 is highly expressed but negative in oral nevi indicating that COX-2 is a valuable marker to distinguish melanocytic lesions of the oral cavity (31), and elevated COX-2 expression is correlated with poor prognosis (32). Similarly, in canine melanoma COX-2 levels are related to Ki-67 proliferation index and survival rate indicating that COX-2 is related to the acquisition of malignancy (7, 16, 33, 34). These results are consistent with our own findings that are presented here; where 89% of all tumours (76 of 85) express some degree of COX-2, with 34% of oral melanomas (10 of 29) and 42% of cutaneous tumours (24 of 56) expressing the highest level of combined distribution and intensity. We also show that COX-2 protein level correlates with Ki-67 proliferation rate, MI and the expression of established melanoma markers.

A mouse model of ovarian cancer has shown a COX-2 gene dosage effect in promoting tumour development, and interestingly they showed that COX-2 may be important for tumour initiation whereas progression and malignancy is driven by COX-1 (35). This is consistent with previous work from our lab showing that COX-2 is overexpressed in the canine osteosarcoma stem cells and that inhibition of COX-2 may be sufficient to inhibit tumour initiation (10). At what times, and in which sub-set of cells, COX-2 expression is required to promote tumourigenesis is unclear. A disadvantage of working with the canine model is a lack of the molecular tools to elucidate the varied roles of COX-2 in tumourigenesis. In this study we describe the creation of two canine melanoma cell lines in which COX-2 expression can be reversibly down-regulated by a doxycycline-inducible miRNA that targets the COX-2 transcript. The vector used has a red fluorescent reporter to allow investigators to assess the feasibility of the inducible system for the cell line specific applications. The ability to reversibly knock-down COX-2 gene expression allows for reversible manipulation of COX-2 expression at any time during tumour progression, in contrast to conventional COX-2 gene disruption strategies which permanently eliminate global COX-2 expression at all times. Here we utilised this system to show that, in the context of canine melanoma, COX-2 is required for cellular proliferation, migration and invasion. Previous studies, consistent with our data, have shown that, by immunohistochemical analysis of COX-2, there is a significant association between COX-2 levels and the proliferation status of cells within a tumour cell population (5, 7, 29, 31). Several studies have also revealed the inhibition of COX-2 by NSAIDs and COXIBs leads to a dose-dependent reduction in cell proliferation in a range of cancer cell lines (15, 36–38). Using CMGD2 and TLM1 cells we have shown that when COX-2 expression is knocked-down there is a significant decrease in cell proliferation and colony forming efficiency compared to the negative control. Although COX-2 has been observed to promote cell proliferation by regulating the activation of downstream oncogenic pathways (39), the underlying molecular mechanism of COX-2 driving cellular proliferation remains to be elucidated.

Melanoma is an aggressive and highly metastatic disease (28). The metastatic process involves the migration of a cell with tumour-initiating potential from the tumour microenvironment followed by the subsequent invasion and colonisation of a secondary site within the body. Here we showed that both the invasive capacity and migration potential of melanoma cells is dependent on COX-2. The epithelial-to-mesenchymal transition (EMT) is a key process involved in acquisition of metastatic potential in number of different types of cancer, including melanoma. During EMT of cancer cells, epithelial cells lose the apical-basal polarity and detach from surrounding cells and transition to invasive mesenchymal cells, which ultimately leads to the active entry of cancer cells into circulation (40). Previous studies have linked COX-2 to the EMT process: COX-2 overexpression has been associated with upregulation of mesenchymal markers and downregulation of epithelial markers (41); and in breast cancer, COX-2 induced production of PGE_2_ inhibited Smad signalling thereby enhancing EMT progression and metastasis (42). Moreover, treatment with various COXIBs led to the reversion of EMT in several cancer cell lines (43, 44). EMT is a key mediator of cancer stem cell plasticity, where by non-cancer stem cells can dedifferentiate and acquire stem cell characteristics, and it is these cells that are responsible for metastatic dissemination. Given that COX-2 has been shown to be overexpressed in cancer stem cells it is interesting to speculate that COX-2 may maintain the cancer stem cell population by driving EMT. The presence of cancer stem cells in different melanoma cell lines and tumours has previously been reported for humans but not for dogs (45–48). To date an evaluation of the role of COX-2 in melanoma stem cell biology has not yet been undertaken. By extension, future research should address the mechanistic role that COX-2/PGE_2_ axis plays in melanoma biology and determining if pharmacological inhibition of COX-2 could prevent cancer stem cell survival, EMT and metastasis.

## Conclusions

Our study provides empirical evidence that COX-2 protein expression is negatively correlated with a worse prognosis in canine patients with melanoma. Extensive clinical studies should be performed to determine the prognostic significance of COX-2 as a marker of canine melanoma. COX-2 is associated with almost all stages of tumour development including tumour initiation, immunosuppression, EMT and metastasis, which all contribute to therapeutic resistance. This is the first study to have developed and characterised a canine-specific DOX-inducible COX-2 miRNA vector. This is a valuable molecular tool for further understanding the mechanisms through which COX-2 effects melanoma progression. Here we have shown that cellular proliferation, invasion and migration are COX-2 dependent in an *in vitro* canine melanoma model. Further studies are required to establish COX-2 as a bona fide therapeutic target in the treatment of canine melanoma.

## Data Availability Statement

The original contributions presented in the study are included in the article/Supplementary Material, further inquiries can be directed to the corresponding author/s.

## Ethics Statement

The animal study was reviewed and approved by Ethics Committee in Animal Experimentation (COMISSÃO DE ÉTICA NO USO DE ANIMAIS/UNIVERSIDADE FEDERAL DE MINAS GERAIS). Written informed consent was obtained from the owners for the participation of their animals in this study.

## Author Contributions

LP wrote the manuscript with support from TS. TS and EF conducted and verified histological classification of patient samples. TS conducted all *in vitro* experiments. AD designed COX-2 miRNA expression vectors. LP and TS performed data analysis. All authors contributed to the article and approved the submitted version.

## Conflict of Interest

The authors declare that the research was conducted in the absence of any commercial or financial relationships that could be construed as a potential conflict of interest.

## Publisher's Note

All claims expressed in this article are solely those of the authors and do not necessarily represent those of their affiliated organizations, or those of the publisher, the editors and the reviewers. Any product that may be evaluated in this article, or claim that may be made by its manufacturer, is not guaranteed or endorsed by the publisher.

## Supplementary Material

Supplementary Figure 1 Panel of negative and positive controls of immunohistochemistry. **(A)** Cutaneous melanoma, negative control, Ki-67 marker. Giemsa counterstain **(B)** Cutaneous melanoma, positive internal control. Ki-67 staining in the nucleus of epidermal cells. DAB staining of chromogen. Giemsa counterstain. **(C)** Cutaneous melanoma, negative control. Melan-A marker. Giemsa counterstain. **(D)** Cutaneous melanoma, positive internal control. Melan-A staining in the cytoplasm of melanocytes cells of the epidermis. DAB staining of chromogen. Giemsa counterstain. **(E)** Cutaneous melanoma. Melanoma Antigen (PNL-2) marker. Giemsa counterstain. **(F)** Cutaneous melanoma. Melanoma Antigen (PNL-2) staining in the cytoplasm of melanocytes cells of the epidermis. DAB staining of chromogen. Giemsa counterstain **(G)** Mucosal Melanoma, negative control. COX-2 marker. Giemsa counterstain. **(H)** Cutaneous melanoma, positive internal control. COX-2 staining in inflammatory cells. DAB staining to chromogen. Giemsa counterstain. All images are objective 40x. Scale bar represents 40 μM.
