# Supplementary figures and images for: COX-2 Silencing in Canine Malignant Melanoma Inhibits Malignant Behaviour
Source: Front Vet Sci. 2021 Aug 26;8:633170. doi: 10.3389/fvets.2021.633170 (PMC8427276; doi:10.3389/fvets.2021.633170)

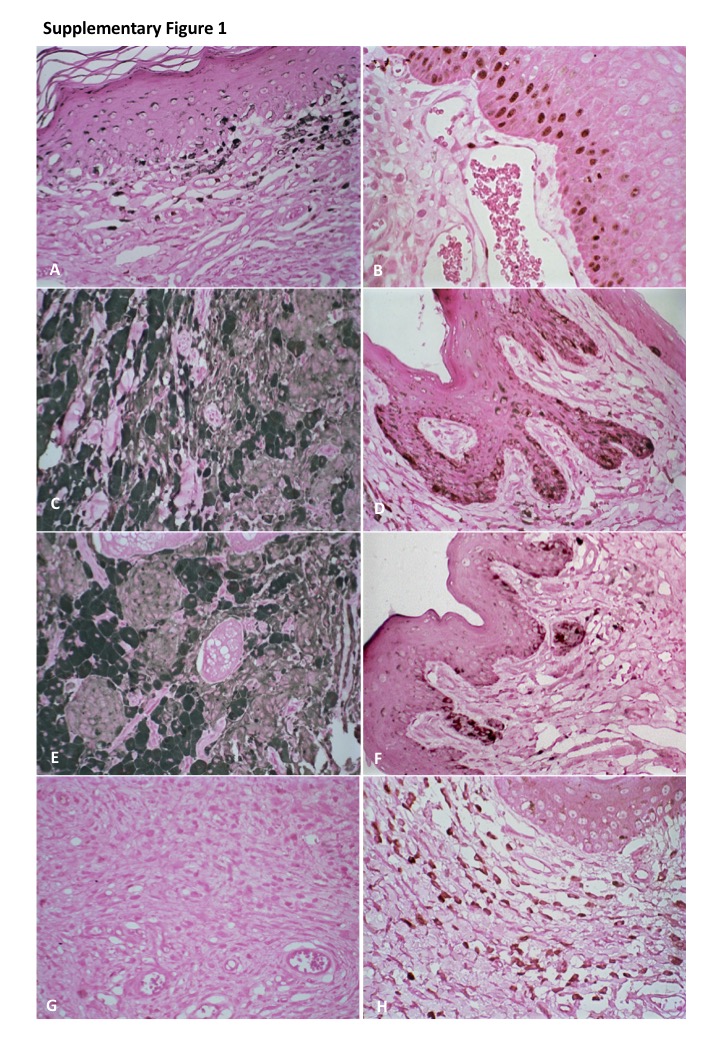

Supplement: Supplementary Figure 1 — Panel of negative and positive controls of immunohistochemistry. (A) Cutaneous melanoma, negative control, Ki-67 marker. Giemsa counterstain (B) Cutaneous melanoma, positive internal control. Ki-67 staining in the nucleus of epidermal cells. DAB staining of chromogen. Giemsa counterstain. (C) Cutaneous melanoma, negative control. Melan-A marker. Giemsa counterstain. (D) Cutaneous melanoma, positive internal control. Melan-A staining in the cytoplasm of melanocytes cells of the epidermis. DAB staining of chromogen. Giemsa counterstain. (E) Cutaneous melanoma. Melanoma Antigen (PNL-2) marker. Giemsa counterstain. (F) Cutaneous melanoma. Melanoma Antigen (PNL-2) staining in the cytoplasm of melanocytes cells of the epidermis. DAB staining of chromogen. Giemsa counterstain (G) Mucosal Melanoma, negative control. COX-2 marker. Giemsa counterstain. (H) Cutaneous melanoma, positive internal control. COX-2 staining in inflammatory cells. DAB staining to chromogen. Giemsa counterstain. All images are objective 40x. Scale bar represents 40 μM. [file Image_1.JPEG]
